# Low back pain patients with Modic type 1 changes exhibit distinct bacterial and non-bacterial subtypes

**DOI:** 10.1016/j.ocarto.2024.100434

**Published:** 2024-01-18

**Authors:** I. Heggli, T. Mengis, C.J. Laux, L. Opitz, N. Herger, D. Menghini, R. Schuepbach, N.A. Farshad-Amacker, F. Brunner, A.J. Fields, M. Farshad, O. Distler, S. Dudli

**Affiliations:** a Center of Experimental Rheumatology, Department of Rheumatology, University Hospital Zurich, University of Zurich, Zurich, Switzerland; b Department of Physical Medicine and Rheumatology, Balgrist University Hospital, Balgrist Campus, University of Zurich, Zurich, Switzerland; c Department of Orthopedics, Balgrist University Hospital, University of Zurich, Zurich, Switzerland; d Functional Genomics Center Zurich, University and ETH Zurich, Zurich, Zurich, Switzerland; e Unit of Clinical and Applied Research, Balgrist University Hospital, University of Zurich, Zurich, Switzerland; f Department of Radiology, Balgrist University Hospital, University of Zurich, Zurich, Switzerland; g Department of Orthopaedic Surgery, University of California San Francisco, San Francisco, CA, USA

**Keywords:** Low back pain, Modic changes, Etiology, *Cutibacterium acnes*, Autoimmunity

## Abstract

**Objectives:**

Modic type 1 changes (MC1) are vertebral endplate bone marrow (BM) lesions observed on magnetic resonance images in sub-populations of chronic low back pain (CLBP) patients. The etiopathogenesis remains unknown and treatments that modify the underlying pathomechanisms do not exist. We hypothesized that two biological MC1 subtypes exist: a bacterial and a non-bacterial. This would have important implications for developing treatments targeting the underlying pathomechanisms.

**Methods:**

Intervertebral disc (IVD) samples adjacent to MC1 (n ​= ​34) and control (n ​= ​11) vertebrae were collected from patients undergoing spinal fusion. *Cutibacterium acnes* (*C.acnes*) genome copy numbers (GCNs) were quantified in IVD tissues with 16S qPCR, transcriptomic signatures and cytokine profiles were determined in MC1 and control BM by RNA sequencing and immunoassay. Finally, we assessed if *C.acnes* GCNs are associated with blood plasma cytokines.

**Results:**

IVD tissues from control levels had <870 ​*C.acnes* GCNs/gram IVD. MC1-adjacent IVDs had either “low” (<870) or “high” (>870) *C.acnes* GCNs. MC1 patients with “high” *C.acnes* GCNs had upregulated innate immune cell signatures (neutrophil, macrophage/monocyte) and pro-inflammatory cytokines related to neutrophil and macrophage/monocyte function in the BM, consistent with a host defense against bacterium. MC1 patients with “low” *C.acnes* GCNs had increased adaptive immune cell signatures (T-and B-cell) in the BM and elevated IL-13 blood plasma levels.

**Conclusion:**

Our study provides the first evidence for the existence of bacterial (*C.acnes* “high”) and non-bacterial (*C.acnes* “low”) subtypes in MC1 patients with CLBP. This supports the need for different treatment strategies.

## 1 Introduction

Low back pain (LBP) is a leading cause of disability [1]. Yet, the pathophysiology remains highly understudied. Modic changes (MC) are vertebral endplate bone marrow (BM) lesions that are visualized as signal intensity changes by magnetic resonance imaging (MRI) [2]. They are prevalent in chronic LBP (CLBP) populations (43 ​% vs. 6 ​% in the asymptomatic population) [12]. MC frequently occur adjacent to a degenerated intervertebral disc (IVDs) and co-locate with vertebral endplate damage [[4], [5], [6], [7], [8], [9], [10]]. Based on their appearance on T1-weighted (T1w) and T2w MR images, three interconvertible types, Modic type 1 changes (MC1), MC2, and MC3, are distinguished. MC1 have the highest predictive value for discography concordant pain and seem to be the most bothersome: MC1 patients suffer from longer and more frequent LBP episodes, seek medical care more often, and are more likely to report little or no improvement in function and pain [[11], [12], [13], [14], [15]]. Treatments that modify the underlying pathomechanisms of MC1 BM lesions do not exist. Clarifying the etiopathogenesis of MC1 may help identify risk factors for MC1 and aid in the development of pathomechanism-modifying treatments.

Prior studies indicate biologic plausibility for bacterial and autoimmune etiologies. Plausibility for a bacterial etiology includes i) the increased MC1 risk if *Cutibacterium acnes* (*C.acnes*), an aerotolerant anaerobic commensal skin bacterium, is present in excised herniated IVD material [16], and by ii) the effectiveness of antibiotic treatment in MC1 patients [[17], [18], [19]]. Furthermore, injecting human IVD-derived *C.acnes* into rat tail IVDs leads to IVD infection, endplate resorption, and MC1-like signal intensity changes on MRI [20]. This suggests that *C.acnes* may invade structurally damaged IVDs and lead to a low-grade IVD infection and occult discitis [4,20]. *C.acnes* proliferation coincides with production of bacterial virulence factors, pro-inflammatory cytokines by IVD cells, and endplate resorption. Subsequent comingling of inflammatory mediators and virulence factors with the adjacent BM leukocytes at sites of endplate damage potentially induce an immune response in the BM [3,4].

Lymphocytic infiltrates in lesions of MC1 patients on the contrary point to an autoimmune MC1 etiology [21]. Implanting IVD cell micro mass pellets into rat tail BM leads to T-cell infiltration around the pellets and provokes MC1-like signal changes on MRI [22,23]. The nucleus pulposus (NP) of the IVD is immune-privileged, resulting from 1) the sequestration of NP cells and matrix after embryologic formation, and 2) from immunological tolerance (the expression of Fas ligand (FASL), which promotes apoptosis in infiltrating lymphocytes). Hence, loss of compartment separation, e.g., through endplate damage, and loss of immune tolerance e.g. through reduced FASL expression [24] could induce an autoimmune response against IVD tissue in the BM [3,4].

Despite the plausibility for bacterial and autoimmune MC1 etiologies, evidence of their existence in clinical populations does not exist. This is because the immune profile of MC1 BM in relation to adjacent intradiscal *C.acnes* genome copy numbers (GCNs) is unknown. We hypothesized that i) MC1 patients have either “low” (similar to degenerated IVDs without adjacent MC) or “high” intradiscal *C.acnes* GCNs, ii) immune cell signatures in the adjacent BM differ between patients with “low” vs. “high” *C.acnes* GCNs, and iii) that these groups have different blood cytokine profiles.

## 2 Materials and methods

### 2.1 Sample collection, imaging, and radiological readouts

BM, IVDs, and blood from CLBP patients (n=38) with and without MC1 undergoing lumbar spinal fusion at the Balgrist University Hospital, Switzerland, between November 2017 and March 2023 were included in this study (Supplementary Table 1). Exclusion criteria were infectious diseases or malignancies. From all patients, IVDs (n=45) were collected under aseptic conditions. From a subset of patients, BM aspirates and blood samples were analyzed. MR images were graded by a radiologist based on available sagittal T1w, T2w, Short Tau Inversion Recovery, and coronal T2w sequences. The mean difference from MRI acquisition to date of surgery was 36.7 ​± ​32.1 days. MC type (MC1, MC2, MC3), total endplate score (0–6) [25] and Pfirrmann degeneration grade (0–5) of IVDs was determined. Samples from patients with pure MC1 (n=14) and mixed MC1-MC2 (n=20) types were included in the analysis.

### 2.2 Intradiscal *C.acnes* GCNs

IVDs (control: n=11; MC1: n=34) were minced, weighted, digested overnight using proteinase K, and mechanically disrupted under aseptic conditions. Bacterial DNA was isolated using the QIAamp UPC Pathogen Mini Kit (Qiagen, Hilden, Germany) according to manufacturer's instructions. *C.acnes* GCNs were quantified with specific primers (Forward: 5′- GCGTGAGTGACGGTAATGGGTA -3′, reverse: 5′-TTCCGACGCGATCAACCA-3′,TaqMan probe 5′-AGCGTTGTCCGGATTTATTGGGCG-3′) using quantitative polymerase chain reaction (qPCR) against a standard of known *C.acnes* GCNs. An additional water sample running through the complete process was included as a contamination control. Median *C.acnes* GCNs between MC1 and control IVDs were compared using Mann-Whitney *U* test, *C.acnes* GCN distributions were compared using Kolmogorov-Smirnov test (RStudio version 4.3.1). The upper 99 ​% confidence interval (CI) limit of the control IVD group was used to stratify and compare BM responses of “high” vs. “low” *C.acnes* groups. To compare demographics between the “high” vs. “low” *C.acnes* GCN groups, patients from whom we collected more than one MC1 IVD (n=3) were classified based on the IVD with highest *C.acnes* GCNs. Continuous parameters were compared between groups with unpaired *t*-test, proportions with Fisher's exact test.

### 2.3 BM aspirate collection

From patients with MC1 (n ​= ​13) two BM aspirates were collected with Jamshidi needles using the pedicle screw trajectories prior to screw insertion. From each patient, a MC1 and an intra-patient control (without any MC) BM aspirate was collected. Aspirates were intraoperatively immediately transferred to K2-EDTA tubes and cells were separated from plasma by centrifugation.

### 2.4 RNA sequencing

For BM total cell bulk RNA sequencing (n ​= ​6), erythrocytes were lysed, 5 million cells were transferred to Qiazol, and RNA was isolated using the miRNeasy Mini Kit (Qiagen, Hilden, Germany) according to manufacturer's instructions. Library was prepared, sequenced and differential expression analysis was performed (Supplementary Methods). Genes were considered to be differentially expressed (DEGs) for p ​< ​0.01. Terms identified with bioinformatic overrepresentation analysis (ORA) and gene set enrichment analysis (GSEA) were considered significantly enriched if false discovery rate (FDR) was <0.05.

### 2.5 BM and blood plasma cytokine measurement

Blood plasma from MC1 patients was obtained like BM plasma. Total protein concentration (pg/ml) of 20 innate and adaptive immunity cytokines (Supplementary Methods) were measured in duplicates in blood and BM plasma with MesoScale U-Plex (Mesoscale Diagnostics). Concentrations of cytokines outside the detection range were set to 0 ​pg/ml (Supplementary Table 2). Blood plasma and normalized BM plasma (Δ_MC1-control_) concentrations were compared between groups with non-parametric Mann-Whitney *U* test and corrected for multiple comparison using Bonferroni correction. Correlations between intradiscal *C.acnes* GCNs and cytokine concentrations were calculated with Spearman correlation. Area Under the Receiver Operating Characteristic Curve (AUC) was computed for each cytokine that correlated with intradiscal *C.acnes* GCNs using simple logistic regression. A logistic regression model was calculated for the combination of cytokines that correlated significantly with intradiscal *C.acnes* GCNs.

### 2.6 Statistical analysis

Statistical analyses were performed using GraphPad Prism version 9.5.1. if not stated otherwise. FDR was calculated in case of multiple comparisons, p-values for single comparisons. Analyses were significant for FDR or p-values<0.05. Normal distribution was tested using the Shapiro Wilk test. Parametric tests were run in case of normal distribution and mean ​± ​standard deviation (SD) is indicated, non-parametric tests in case of non-normal distribution where median, [interquartile range (IQR)] is given.

## 3 Results

### 3.1 Patient characteristics

Patients had an average age of 64.1 ​± ​11.2 years, and 60.5 ​% were female. Fifty percent of the patients smoked. Patients were on average overweight (body mass index (BMI): 28.2 ​± ​5.5 ​kg/m^2^), had high back-(visual analogue score (VAS)_back_ ​= ​7.2 ​± ​1.9) and leg pain (VAS_leg_ ​= ​6.6 ​± ​2.7) and high disability (Oswestery disability index (ODI): 46.3 ​± ​15.2 ​%). Seven of the 38 patients (18.4 ​%) had prior invasive back surgery (46.1 ​± ​35.5 months before surgery), 30 patients (79.0 ​%) obtained at least once an epidural infiltration (9.7 ​± ​9.9 months before surgery), and 3 patients (7.8 ​%) had a prior facet joint infiltration (18.3 ​± ​11.2 months prior surgery).

### 3.2 Intradiscal *C.acnes* distribution in MC1 and control IVDs

**Fig. 1:**
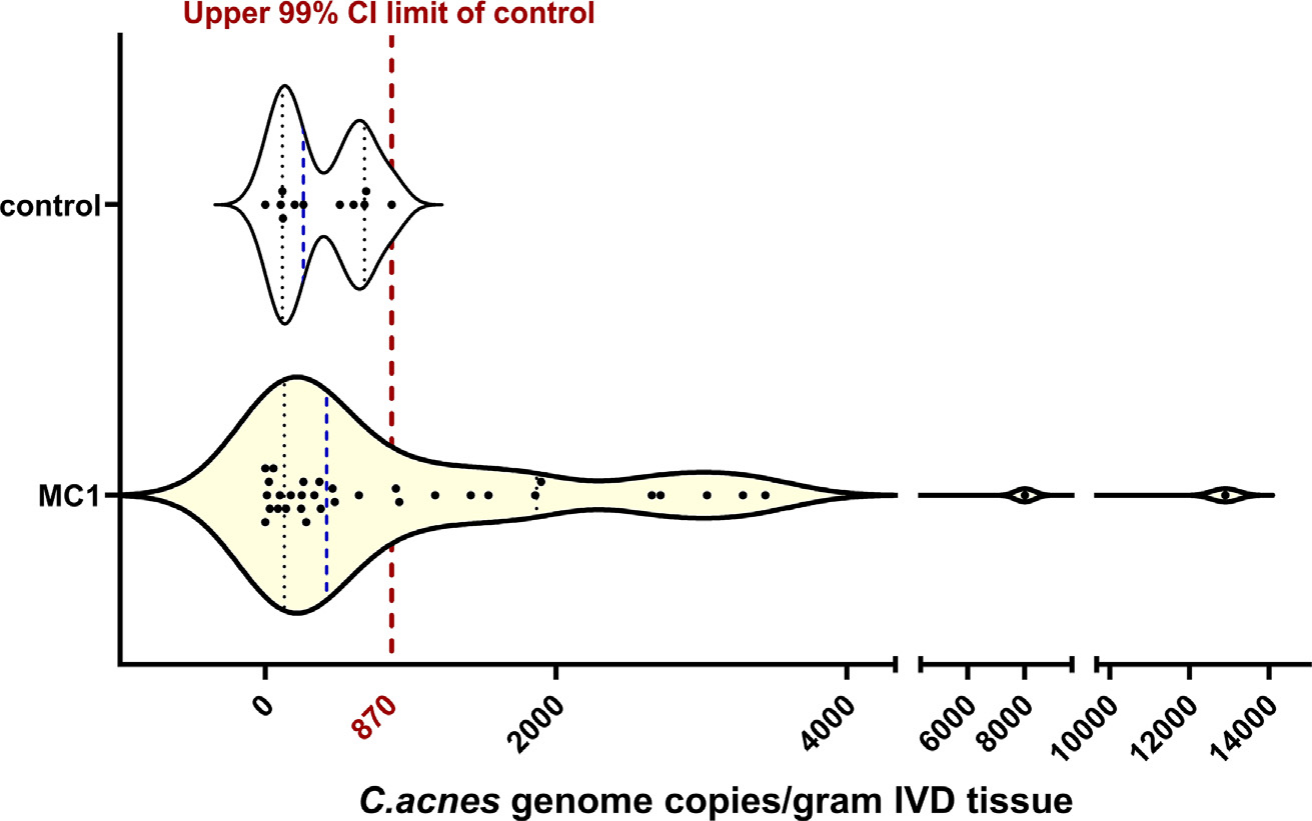
*C.acnes* copy number distribution of control (upper plot) and MC1 (lower plot) intervertebral discs (IVDs). Blue line: Median *C.acnes* GCNs/gram IVD tissue; Red line: Upper 99 ​% confidence interval limit of the control IVDs. Ns ​= ​Not significant. Control: n ​= ​11, MC1: n ​= ​34.

From 38 patients, 45 IVDs (control: n ​= ​11, MC1: n ​= ​34) were collected (one sample MC1 IVDs: n ​= ​25 patients; one sample control IVDs: n ​= ​7 patients; two sample MC1 and control IVDs: n ​= ​3 patients; two sample MC1 IVDs: n ​= ​3 patients). Pfirrmann grade did not differ between control and MC1 IVDs (control: 4.5,[4.0,5.0]; MC1: 5.0,[4.0,5.0], p ​= ​0.36), but endplates were significantly more degenerated in MC1 (control: 3.0,[3.0,5.3]; MC1: 6.0,[5.0,6.0], p ​= ​0.009). None of the control IVDs exceeded 870 ​*C.acnes* GCNs/gram IVD (median: 262,[117,682]; 99 ​% CI (0, 870)) (Fig. 1). Overall, there was no statistical difference (p ​= ​0.29) in median *C.acnes* GCNs between control and MC1 IVDs (n ​= ​34) (423,[132,1866]; p ​= ​0.28), however, there was a significant difference (D ​= ​0.411, p ​= ​0.04) in *C.acnes* GCN distribution between control and MC1 IVDs with 14 MC1 IVDs (38.9 ​%) having >870 GCNs/gram IVD (Fig. 1). Since we hypothesized that a MC1 subtype with similar *C.acnes* GCNs like degenerated IVDs without adjacent MC1 exists, we defined the subtype separation threshold as the upper 99 ​% CI limit of the control group (870 GCNs/gram IVD). Dichotomizing the MC1 group based on this threshold resulted in a *C.acnes* “low” group (211,[37,364] GCNs/gram IVD) with similar GCNs as control IVDs, and a *C.acnes* “high” group (2278,[1352,3324]), whose GCNs were 10.8-fold higher than the *C.acnes* “low” group, on average.

MC1 patients of the *C.acnes* “low” (n ​= ​17) and “high” (n ​= ​14) groups did not differ in age, BMI, disability, back and leg pain, IVD and endplate degeneration, nor in the proportions of females, smokers, patients with prior back surgery/epidural- or facet joint infiltrations (Supplementary Table 3).

### 3.3 Transcriptomic changes in MC1 BM cells

**Fig. 2:**
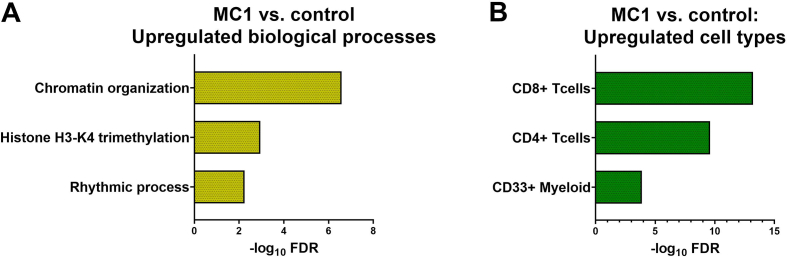
Transcriptomic signatures of MC1 BM cells. Top three significantly enriched biological processes (BPs) (A) and cell types (B) identified by ORA of upregulated differentially expressed genes (DEGs) are shown. MC1: n ​= ​6, intra-patient controls: n ​= ​6.

Six MC1 patients were selected for BM cell RNA sequencing. Overall, these patients had a median of 505,[22,3138] *C.acnes* GCNs/gram IVD. Three patients belonged to the *C.acnes* “low” group and had on average 25,[11,87]) GCNs/gram IVD. The other three patients belonged to the *C.acnes* “high” group and had on average 3038,[922,3439] GCNs/gram IVD. Comparing MC1 to intra-patient control irrespective of adjacent *C.acnes* GCNs revealed 333 DEGs. ORA of the upregulated DEGs showed enriched biological processes (BPs) associated with chromatin remodeling (Fig. 2A) in MC1 BM cells. The top upregulated cell types in MC1 were T-cells (CD8^+^, CD4^+^), myeloid cells, and natural killer cells (NKCs), cells belonging to the adaptive (T-cells) and innate (myeloid cells, NKCs) immune system (Fig. 2B). Pathway analysis revealed a trend towards upregulated adipogenesis related pathways like “Transcriptional regulation of white adipocyte differentiation” (FDR ​= ​0.08, p-value ​= ​2.9E-5) and “Lipid metabolism regulation by PPAR-alpha” (FDR ​= ​0.08, p-value ​= ​3.6E-5) as well as “B-cell activation” (FDR ​= ​0.18, p-value ​= ​4.5E-4). Without stratifying MC1 patients, transcriptomic changes of MC1 BM cells showed enriched adaptive and innate immune system processes in the BM.

**Fig. 3:**
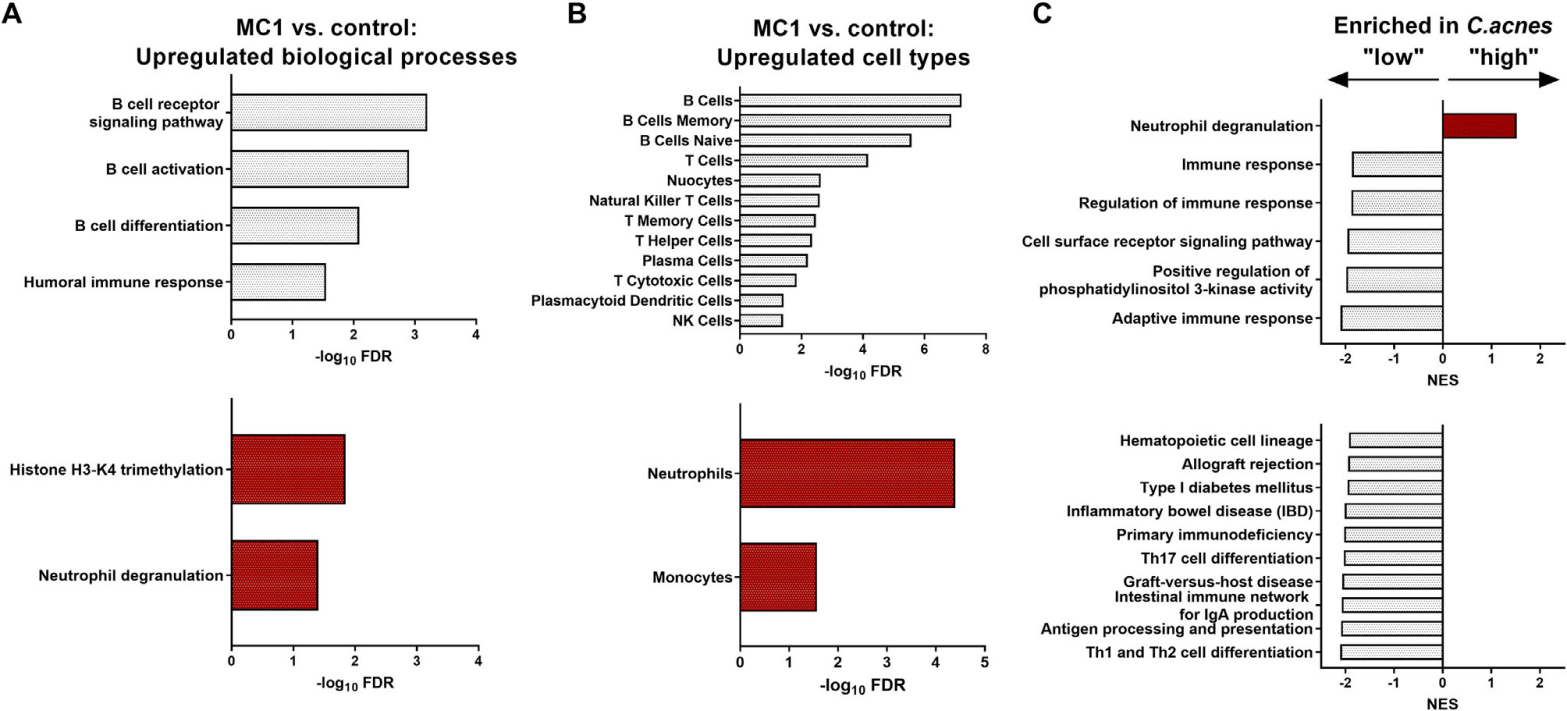
Bone marrow (BM) cell bulk RNA sequencing. Upregulated biological processes (BPs) (A) and cell types (B) in MC1 vs. intra-patient control total BM cells in *C.acnes* “low” (top) and *C.acnes* “high” (bottom) groups identified by ORA. (C) Top enriched BPs (top) and pathways (bottom) comparing total BM cells between *C.acnes* “low” vs. “high” group identified by gene set enrichment analysis. *C.acnes* “low”: MC1: n ​= ​3, intra-patient controls: n ​= ​3. *C.acnes* “high”: MC1: n ​= ​3, intra-patient controls: n ​= ​3.

When stratifying patients into *C.acnes* “high” and “low” GCN groups, differential expression analysis revealed 79 DEGs in the *C.acnes* “low” group and 352 DEGs in the *C.acnes* “high” group between MC1 and intra-patient control BM. ORA showed that all upregulated BPs in MC1 of the *C.acnes* “low” group were related to B-cell processes. In comparison, chromatin remodeling and neutrophil degranulation were upregulated in *C.acnes* “high” MC1 patients (Fig. 3A). B-and T-cells, cells belonging to the adaptive immune system were the top upregulated cell types in the *C.acnes* “low” group. In the *C.acnes* “high” group, innate immune cells (neutrophils/monocytes) were upregulated (Fig. 3B). The top enriched BPs comparing *C.acnes* “low” to “high” groups were “adaptive immune response” (FDR ​= ​0.00) in *C.acnes* “low” compared to “neutrophil degranulation” (FDR ​= ​0.00) in the *C.acnes* “high” group. GSEA of pathways further showed significantly enriched T helper subset differentiation pathways in *C.acnes* “low” MC1 patients like “Th1 and Th2 cell differentiation” (FDR ​= ​0.00) and “Th17 ​cell differentiation” (FDR ​= ​0.00) (Fig. 3C). In summary, transcriptomic analysis of MC1 BM cells revealed upregulated adaptive (B-, T-cells) immunity signatures in *C.acnes* “low” MC1 BM and innate immunity (neutrophils/monocytes) in the *C.acnes* “high” BM.

### 3.4 BM and blood plasma cytokine profiles

**Fig. 4:**
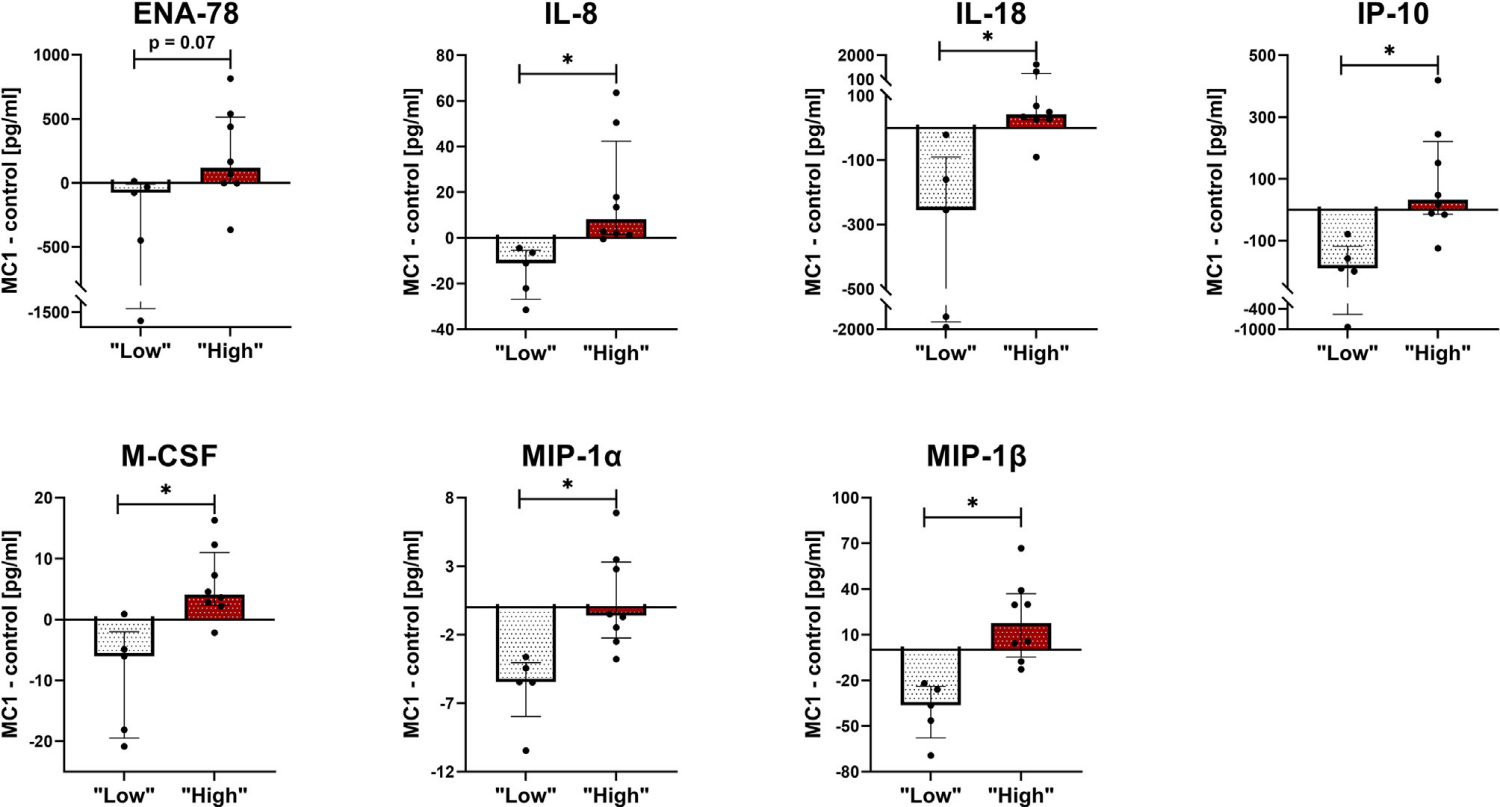
*C.acnes* “low” and “high” group bone marrow (BM) plasma cytokine profiles. (A) Δ_MC1-control_ BM protein concentrations (pg/ml) of *C.acnes* “low” (white bars) and “high” (red bars) groups. Figure shows cytokines with significant and trend towards significant (ENA-78) inter-group differences. Bars represent median Δ_MC1-control_ BM protein concentrations, error bars indicate interquartile range. *C.acnes* “low”: MC1: n ​= ​5, intra-patient controls: n ​= ​5. *C.acnes* “high”: MC1: n ​= ​8, intra-patient controls: n ​= ​8. ∗ FDR<0.05.

Twenty cytokines associated with innate and adaptive immune pathways were quantified in 13 MC1 and intra-patient control BM plasma (*C.acnes* “low”: 25,[6175] GCNs/gram IVD, n ​= ​5; *C.acnes* “high”: 3162,[1542,6870] GCNs/gram IVD, n ​= ​8). Comparing intra-patient cytokine differences (Δ_MC1-control_) between “high” vs. “low” *C.acnes* GCN groups revealed increased epithelial-neutrophil activating peptide (ENA-78) (FDR ​= ​0.07), interleukin (IL)-8 (FDR ​= ​0.001), IL-18 (FDR ​= ​0.01), interferon gamma-induced protein 10 (IP-10) (FDR ​= ​0.01), macrophage colony stimulating factor (M-CSF) (FDR ​= ​0.01), macrophage inflammatory proteins (MIP)-1α (FDR ​= ​0.01), and MIP-1β (FDR ​= ​0.01) cytokine levels in the *C.acnes* “high” MC1 group (Fig. 4, Supplementary Table 4). Furthermore, intradiscal *C.acnes* GCNs correlated positively with BM cytokine concentrations of IL-1β (ρ ​= ​0.61, p-value ​= ​0.039), IL-8 (ρ ​= ​0.72, p-value ​= ​0.007), IL-18 (ρ ​= ​0.78, p-value ​= ​0.003), IP-10 (ρ ​= ​0.74, p-value ​= ​0.005), M-CSF (ρ ​= ​0.74, p-value ​= ​0.006), MIP-1α (ρ ​= ​0.79, p-value ​= ​0.002), and MIP-1β (ρ ​= ​0.77, p-value ​= ​0.003), and negatively with granulocyte colony stimulating factor (G-CSF) (ρ ​= ​−0.70, p-value ​= ​0.01) (Supplementary Table 5). ENA-78 and IL-8 attract and activate neutrophils. M-CSF promotes macrophage differentiation. MIP-1α, MIP-1β, IL-18, IL-1β, IP-10 are pro-inflammatory cytokines/chemokines predominantly produced by macrophages/monocytes under inflammatory conditions. Taken together, pro-inflammatory cytokines/chemokines related to neutrophil and macrophages/monocyte function or production were found to be increased in the BM plasma of the *C.acnes* “high” MC1 patients.

**Fig. 5:**
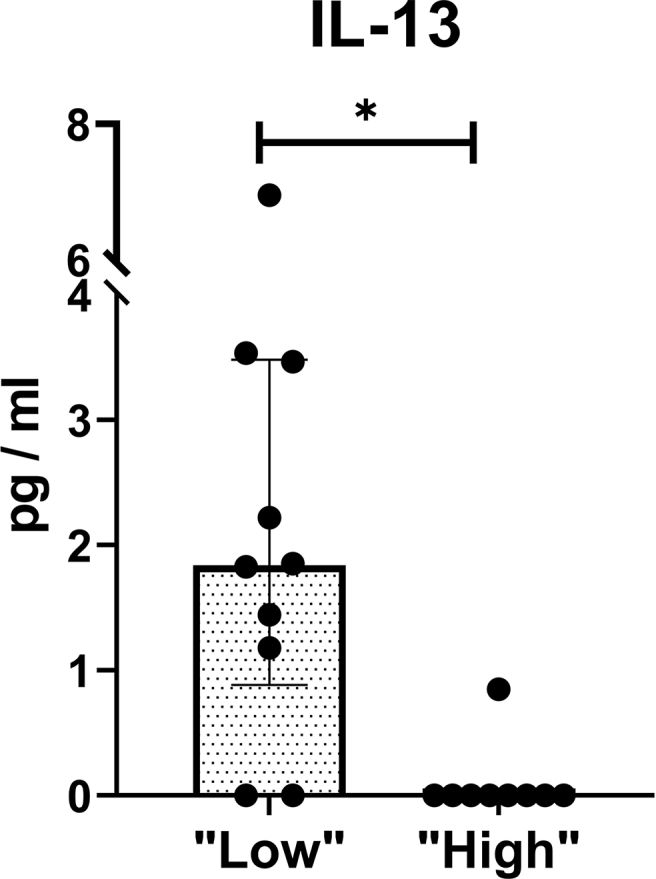
*C.acnes* “low” and “high” MC1 group blood plasma cytokine profile. Figure shows significantly different (FDR<0.05) cytokines between groups. Bar represent median blood protein concentrations, error bars indicate interquartile range. *C.acnes* “low”: n ​= ​10; *C.acnes* “high”: n ​= ​9.∗ P-value<0.05.

The same 20 cytokines were measured in the blood plasma of *C.acnes* “low” (181,[22,375] GCNs/gram IVD, n ​= ​10) and “high” (1897,[1228,5650], GCNs/gram IVD, n ​= ​9) MC1 patients. IL-13, a cytokine found to be increased in patients with autoimmune diseases [26,27], was increased in MC1 patients with “low” compared to “high” intradiscal *C.acnes* GCNs (FDR ​= ​0.02) (Fig. 5, Supplementary Table 6). In the *C.acnes* “high” group, IL-13 was only detectable in 1 of 9 patients (11 ​%), whereas it was detected in 8 of 10 patients (80 ​%) in the *C.acnes* “low” group. Furthermore, IFN-γ (ρ ​= ​−0.64, p-value ​= ​0.004), IL-12p70 (ρ ​= ​−0.54, p-value ​= ​0.018), and IL-13 (ρ ​= ​−0.71, p-value ​= ​0.0006) correlated negatively with intradiscal *C.acnes* GCNs in the MC1 IVD (Supplementary Table 7). IL-13 distinguished best between the two groups (AUC ​= ​0.89, sensitivity ​= ​100 ​%, specificity ​= ​70 ​%). In combination with IFN-γ and IL-12p70, the discriminatory ability was further increased (AUC ​= ​0.92, sensitivity ​= ​89 ​%, specificity ​= ​80 ​%) (Table 1).

**Table 1:** Receiver Operating Characteristics (ROC) of cytokines that discriminated best between *C. acnes* “low” and “high” MC1 groups. Area under the curve (AUC) calculated by simple logistic regression for individual cytokines (top three rows) and by logistic regression model (bottom row) of cytokine combination. CI: Confidence interval.

Cytokine	AUC	95 % CI	Sensitivity	95 % CI	Specificity	95 % CI	P-Value
IFN-y	0.83	(0.64, 1.00)	100 ​%	(70, 100)	70 ​%	(40,89)	0.01
IL-12p70	0.79	(0.57, 1.00)	89 ​%	(57,99)	80 ​%	(49, 96)	0.03
IL-13	0.89	(0.72, 1.00)	100 ​%	(70, 100)	80 ​%	(49, 96)	0.00
IFN-y ​+ ​IL-12p70 ​+ ​IL-13	0.92	(0.79, 1.00)	89 ​%	(68, 100)	80 ​%	(55, 100)	0.00

## 4 Discussion

MC1 are vertebral endplate BM lesions frequently observed on MRI in CLBP patients. Yet, the pathobiology remains poorly understood and treatments that modify the underlying pathomechanisms do not exist. A major obstacle is the unclear existence of different subtypes in MC1 patients. Here, we performed the first in-depth characterization of MC1 BM. We found evidence for the existence of two biological MC1 subtypes, (i) an intradiscal “high” *C.acnes* GCN subtype with an activation of the innate immune system in the MC1 BM, and (ii) an intradiscal “low” *C.acnes* GCN subtype with an activation of the adaptive immune system in the MC1 BM. Finally, we found blood plasma cytokines that were able to distinguish these subtypes with high accuracy. This has important clinical implications because different subtypes may require different treatments.

BM inflammation has been attributed as a hallmark of MC1 [2,21,28]. However, evidence for inflammation is poor and inflammatory pathomechanisms are largely unexplored. Upregulated chromatin remodeling and H3K4 trimethylation underscore inflammation to be a MC1-specific hallmark, because chromatin is remodeled in response to pro-inflammatory cytokines and leads to the production of pro-inflammatory mediators itself, and increased H3K4 trimethylation correlates with an upregulation of genes involved in inflammation [29,30]. Even though fatty marrow replacement is traditionally attributed to MC2, we found upregulated adipogenic signatures in MC1 BM. Adipose tissue itself can be pro-inflammatory [31]. Hence, adipogenesis could be a yet undiscovered MC1-specific inflammatory process. Interestingly, a link between increased H3K4 trimethylation and adipogenesis has been shown [30]. Whether this link also exists in MC1 BM and if adipogenesis indeed is an inflammatory MC1 pathomechanism needs to be further investigated. Furthermore, our data show that both adaptive (T-, B-cell) and innate (myeloid-, and NKC) arms of the immune system seem to contribute to BM inflammation in MC1. Overall, our analysis supports inflammation as a MC1-specific hallmark and unravels unexplored potential inflammatory pathomechanisms.

There is an ongoing debate whether different subtypes exist in MC1 patients. Here, we provide the first evidence for *C.acnes* “high” and “low” MC1 subtypes, which suggests the existence of predominant bacterial (*C.acnes* “high”) and non-bacterial (potentially autoimmune) (*C.acnes* “low”) MC1 subtypes.

Our finding that only MC1 but not control IVDs have “high” *C.acnes* GCNs supports two biological subtypes in MC1. Even though the overall median *C.acnes* GCNs are similar in MC1 and control IVDs, the distributions differ significantly from each other, which supports a bacterial and a non-bacterial MC1 subtype.

“High” intradiscal *C.acnes* GCNs and upregulated innate immunity (neutrophil, monocycte/macrophage) transcriptomic signatures and cytokines in the BM *s* provide evidence for a predominant bacterial (*C.acnes*-mediated) MC1 subtype. The finding that only MC1 but not control IVDs have high *C.acnes* GCNs suggests ongoing or past bacterial proliferation in this group. Neutrophils and monocytes/macrophages together orchestrate a complex response to eliminate pathogens. Neutrophil degranulation was the top enriched process comparing the BM of *C.acnes* “high” to “low” MC1 patients. The granular content of neutrophils has high antimicrobial activity and neutrophil degranulation is a well-known mechanism to fight bacterial infections [32]. Neutrophils become attracted and activated to release granules for example by ENA-78 and IL-8 [33,34], both cytokines we found to be upregulated in *C.acnes* “high” MC1 BM. This could also explain the predominant neutrophil signatures. The production site of these cytokines needs to be investigated, but IL-8 could originate from the adjacent IVD. IVDs adjacent to MC produce more IL-8 [28,35]. Interestingly, NP cells secrete increased levels of IL-8 upon *in vitro* stimulation with *C.acnes* [36]. Thus, we hypothesize that IL-8 produced in the IVD as a response to *C.acnes* may have drained through damaged endplates into the adjacent BM, thereby attracting and activating adjacent BM neutrophils. In support of this hypothesis, biological crosstalk between the IVD with the adjacent BM has been shown [28]. Besides evidence for a neutrophil-mediated antimicrobial response, transcriptomic signatures and cytokine analysis further unraveled upregulated monocyte/macrophage signatures, another innate immunity cell type critical in fighting bacteria. Besides being involved in inflammation and adipogenesis, upregulation of H3K4 trimethylation was shown to be an epigenetic activation marker of monocytes in response to bacterial pathogens [37]. This is further indicative for an antimicrobial specific response in *C.acnes* “high” MC1 BM. Furthermore, upregulated M-CSF indicates recruitment of monocytes/macrophages and it was shown that monocytes/macrophages produce MIP-1α, MIP-1β, IL-18, and IP-10 in response to bacteria [[38], [39], [40]]. All these cytokines were found to be upregulated in *C.acnes* “high” MC1 BM. Overall, “high” intradiscal *C.acnes* GCNs and upregulated innate immunity signatures and cytokine profiles associated with a defense response against bacteria supports the existence of a bacterial (*C.acnes*-mediated) MC1 biological subtype.

Evidence that MC1 patients with “low” intradiscal *C.acnes* GCNs represent patients of a non-bacterial (potentially autoimmune) MC1 subtype is supported by similar *C.acnes* GCNs that control IVDs, upregulated adaptive immunity signatures, and blood cytokines found to be associated with autoimmune disorders. T- and B-cells become activated upon exposure to IVD tissue and it has been shown that autoantibodies against IVD extracellular matrix proteins can be produced [41,42]. Patients of the *C.acnes* “low” GCN group have upregulated B- and T-cell signatures, which could indicate an adaptive (potentially autoimmune) immune response against the IVD. Th1/Th2- and Th17 differentiation were among the most enriched pathways in this group, all of which play a pivotal role in the autoimmunity of many rheumatic diseases [[43], [44], [45]]. Adaptive immunity itself is not an indication for autoimmunity, which would need to be confirmed with presence of autoantibodies. However, it shows that critical players in autoimmune responses are upregulated in *C.acnes* “low” patients, which supports a non-bacterial (potentially autoimmune) MC1 subtype. The blood cytokine analysis of our study provides further evidence that the BM inflammation in the *C.acnes* “low” MC1 group could reflect an autoimmune response against the IVD. IL-13 was significantly upregulated in *C.acnes* “low” MC1 patients, a cytokine also found to be increased in the serum of patients with (Th2-mediated) autoimmune diseases like systemic lupus erythematosus and systemic sclerosis. [26,27,46]. The role of IL-13 in the pathogenesis of autoimmune disorders is still not fully understood, but a role in T-helper cell dysregulation is suggested. Nevertheless, increased IL-13 blood plasma levels in MC1 patients with “low” *C.acnes* GCNs is further supportive for a non-bacterial (potentially autoimmune) MC1 subtype. Interestingly, IL-13 in combination with IFN-y and IL-12p70, cytokines also associated with autoimmune responses, had high accuracy to discriminate between *C.acnes* “low” and “high” MC1 patients. How these blood cytokines associate with immunological responses in the BM and if they would be suitable biomarkers for patient stratification needs to be shown. Taken together, our data provide evidence for the existence of a bacterial and a non-bacterial subtype in MC1 patients.

Demonstrating the existence of different MC1 subtypes addresses an unmet clinical need because different subtypes may require different treatment strategies. To develop subtype-specific treatments, it is essential to develop a diagnostic tool to stratify patients of different MC1 subtypes. Blood biomarkers that reflect the intradiscal *C.acnes* GCNs would be beneficial, since *C.acnes* quantification in IVDs require removal of IVD material or analysis of BM plasma/cells. Bråten et al. (2019) found no predictive effect of 40 inflammatory serum cytokine levels on amoxicillin treatment in LBP patients with and without MC1 [47]. In concurrence with their finding, the only cytokine that differed significantly between our groups was IL-13, a cytokine they did not measure. They also did not measure IL-12p70, which with IFN-y together increased the discriminatory ability between *C.acnes* “high” and “low” groups. It is important to state that we did not perform a biomarker study, but it was the aim to show the existence of biological MC1 subtypes. If IL-12p70, IL-13, and IFN-y plasma levels could serve as potential subtype separation biomarkers needs to be addressed in a larger biomarker study. Patient stratification is likely critical for successful treatment outcomes in the future and should also be considered for future clinical trials. For example, MC1 patients of the *C.acnes* “high” subtype may respond better to antibiotic treatment, whereas intradiscal steroid usage might even be detrimental. Our evidence for the existence of distinct MC1 biological subtypes could also explain inconsistencies in the outcome of currently used treatment modalities for MC1 (intradiscal steroids vs. antibiotics) [17,18,48,49]. Hence, our study strongly supports that biological subtypes in MC1 patients indeed exist, which has large implications on future diagnostic tools and treatment strategies.

One limitation of this study is the inability to investigate whether biological MC1 subtypes represent different stages of the same pathology. Collection of IVD and adjacent BM biospecimens of MC1 patients over time is not feasible and would need to be studied in animal models or quantified non-invasively with MR spectroscopy [50]. Hence, we cannot draw conclusions about the MC1 etiology and we here refer to biological subtypes. Moreover, bacterial contamination during IVD collection and processing cannot be excluded. However, we focused on keeping sterile conditions throughout biomaterial processing and potential contaminations would have affected both MC1 control IVDs. Importantly, biological findings in the BM and peripheral blood could not have been influenced by processing contamination. The upper 99 ​% CI limit of control IVDs was chosen as subtype separation threshold assuming that i) at least two MC1 subtypes exist, ii) all degenerated IVDs contain some *C.acnes* GCNs, and iii) a certain *C.acnes* GCN is required to induce adjacent MC1 BM lesions. Moreover, biological reactions are likely not on/off as suggested with dichotomizing but rather gradually. Hence, this threshold should not be considered as an absolute threshold, may vary across studies, and needs to be confirmed in another cohorts. Furthermore, *C.acnes* GCNs do not give indications about bacterial viability, pathogenicity, or if the bacteria stems from the IVD or if it was introduced due to contamination. The sample size for the BM analyses were low and the results need to be interpreted with caution. However, the usage of intra-patient control samples enhances statistical power and allows to minimize inter-patient variability. Blood cytokine levels were not compared with age – and sex-matched healthy controls and LBP patients with no-MC. To evaluate, if the combination of IL-12p70, IL-13, and IFN-y could be used as potential subtype-specific peripheral blood biomarkers, future biomarker studies in different cohorts need to be performed.

In conclusion, we show that *C.acnes* “high” and “low” subtypes exist in MC1 patients that have distinct immunological BM signatures. This supports the evidence for the existence of bacterial (*C.acnes*-mediated) and non-bacterial subtypes in MC1 patients. This has large clinical implications, since patients of different etiologies may require treatment strategies.

## Author contributions

Study conception and design: IH, SD, OD, AJF.

Acquisition of data: IH, TM, CJL, NH, DM, RS, FB, MF, SD.

Analysis and interpretation of data: IH, TM, CJL, LO, NH, DM, NAF, AJF, OD, SD.

Drafting/revising article and approving of final version: All authors.

Funding: SD.

## Role of the funding source

This work was supported by the VELUX foundation (1170), Balgrist foundation, CRPP Pain foundation.

## Financial support

VELUX foundation (1170), Balgrist foundation, CRPP Pain.

## Declaration of competing interest

OD has/had consultancy relationship with and/or has received research funding from and/or has served as a speaker for the following companies in the area of potential treatments for systemic sclerosis and its complications in the last three calendar years: 4P-Pharma, AbbVie, Acceleron, Alcimed, Altavant, Amgen, AnaMar, Argenx, Arxx, AstraZeneca, Blade, Bayer, Boehringer Ingelheim, Corbus, CSL Behring, Galderma, Galapagos, Glenmark, Gossamer, Horizon, Janssen, Kymera, Lupin, Medscape, Merck, Miltenyi Biotec, Mitsubishi Tanabe, Novartis, Prometheus, Redxpharma, Roivant and Topadur. Patent issued “mir-29 for the treatment of systemic sclerosis” (US8247389, EP2331143). Co-founder of CITUS AG.

SD is an inventor of the patent of Aclarion.
