# Supplementary material for: Low back pain patients with Modic type 1 changes exhibit distinct bacterial and non-bacterial subtypes
Source: Osteoarthr Cartil Open. 2024 Jan 18;6(1):100434. doi: 10.1016/j.ocarto.2024.100434 (PMC10844677; doi:10.1016/j.ocarto.2024.100434)
Supplement: Multimedia component 1 [file mmc1.docx]

**Supplementary tables**

**Supplementary Table 1:** Sample overview.

|  | **Intervertebral discs** | **Bone marrow** | | **Blood** |
| --- | --- | --- | --- | --- |
|  |  | Cells | Plasma | Plasma |
|  | 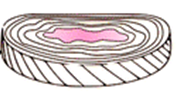 | 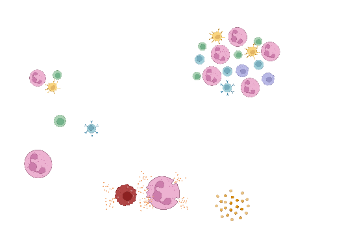 | 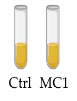 | 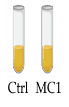 |
|  |  |  |  |  |
|  |  |  |  |  |
| **Overall** |  |  |  |  |
| MC1 | 34 | 6 | 13 | 19 |
| No MC1 | 11 | 6 | 13 |  |
| **Paired analyses** |  |  |  |  |
| IVD - bone marrow cells |  | 6 |  |  |
| IVD - bone marrow plasma |  |  | 13 |  |
| IVD - blood plasma |  |  |  | 19 |

**Supplementary Table 2:** Cytokine curve fit limits measured with Mesoscale UPlex Discovery Assay. Top row shows curve fit limits per cytokine. Middle (bone marrow) and bottom (blood) rows indicate how many samples were not detected per cytokine and separated in *C.acnes* “high” and *C.acnes* “low” groups.

|  |  | **GM-CSF** | **IFN-y** | **IL-1b** | **IL-4** | **IL-6** | **IL-8** | **IL-10** | **IL-12-p70** | **IL-13** | **IL-17A** | **ENA-78** | **G-CSF** | **IL-18** | **IL-7** | **IP-10** | **MCP-1** | **M-CSF** | **MIP-1a** | **MIP-1b** | **TNF-a** |
| --- | --- | --- | --- | --- | --- | --- | --- | --- | --- | --- | --- | --- | --- | --- | --- | --- | --- | --- | --- | --- | --- |
|  | Curve fit limits (pg/ml) | 0.02-9400 | 1.7-17000 | 0.15-3800 | 0.02-2100 | 0.33-2000 | 0.15-2200 | 0.08-3700 | 0.05-5300 | 0.85-1900 | 0.34-23400 | 0.53-3900 | 1.60-20400 | 0.5-14000 | 1.30-7000 | 0.49-6000 | 0.74-6600 | 0.29-2000 | 7.70-4200 | 1.50-1600 | 0.51-3700 |
| **Bone marrow** (number of samples out of limit) | C.acnes "low" |  |  |  |  |  |  | 2 | 3 | 3 | 2 |  |  |  |  |  |  |  |  |  |  |
|  | C.acnes "high" |  |  | 1 |  |  |  | 2 | 2 |  | 2 |  |  |  |  |  |  |  |  |  |  |
| **Blood** (number of samples out of limit) | C.acnes "low" | 6 |  | 7 | 4 |  |  | 1 | 2 | 8 | 2 |  |  |  | 1 | 1 |  |  |  |  |  |
|  | C.acnes "high" | 4 |  | 6 | 3 |  |  | 1 | 1 | 2 | 1 |  |  |  |  |  |  |  |  |  |  |

**Supplementary Table 3:** Demographics of patients with “high” and “low” intradiscal *C.acnes* load.

|  | **Age [years]** | **BMI [kg/m2]** | **ODI [%]** | **VAS back** | **VAS leg** | **DD** | **TES** | **Females [%]** | **Smokers [%]** | **Prior back surgery [%]** | **Prior epidural infiltrations [%]** | **Prior facet joint infiltrations [%]** |
| --- | --- | --- | --- | --- | --- | --- | --- | --- | --- | --- | --- | --- |
| ***C. acnes* "low"** | 64.6 ± 12.9 | 29.3 ± 5.2 | 45.4 ± 13.3 | 7.7 ± 1.5 | 7.0 ± 2.5 | 5.0, [4.0, 5.0] | 6.0, [4.3, 6.0] | 52.9 | 58.8 | 12.5 | 76.5 | 10 |
| ***C. acnes* "high"** | 64.7 ± 10.3 years | 26.5 ± 4.7 | 41.9 ± 13.3 | 7.0 ± 0.6 | 5.8 ± 2.6 | 5.0, [5.0, 5.0] | 6.0, [5.0, 6.0] | 57.1 | 35.7 | 23.1 | 77 | 7.8 |
| **P-value** | 0.98 | 0.14 | 0.51 | 0.26 | 0.22 | 0.4 | 0.97 | 0.55 | 0.18 | 0.4 | 0.67 | 0.67 |

**Supplementary Table 4:** Δ_MC1-control_ bone marrow (BM) plasma cytokine profiles of *C.acnes* “low” and “high” MC1 patients. MC1 patients of “low” group: n=5; MC1 patients of “high” group: n=8.

|  |  | **GM-CSF** | **IFN-y** | **IL-1b** | **IL-4** | **IL-6** | **IL-8** | **IL-10** | **IL-12-p70** | **IL-13** | **IL-17A** | **ENA-78** | **G-CSF** | **IL-18** | **IL-7** | **IP-10** | **MCP-1** | **M-CSF** | **MIP-1a** | **MIP-1b** | **TNF-a** |
| --- | --- | --- | --- | --- | --- | --- | --- | --- | --- | --- | --- | --- | --- | --- | --- | --- | --- | --- | --- | --- | --- |
| ***C.acnes* "low"  (n = 3-5)** | Median | 0.00 | 0.73 | -0.66 | -0.04 | 2.59 | -11.06 | 0.27 | 0.08 | -1.15 | 0.07 | -76.15 | 1.62 | -255.64 | -0.08 | -189.08 | 17.30 | -6.04 | -5.45 | -36.31 | -0.58 |
|  | 1. Quartile | -0.13 | -0.06 | -1.08 | -0.09 | -0.47 | -22.06 | -0.21 | -0.02 | -1.40 | -0.08 | -449.58 | 1.26 | -1211.01 | -2.45 | -198.97 | -52.11 | -18.12 | -5.47 | -46.32 | -0.92 |
|  | 3. Quartile | 0.05 | 2.95 | -0.58 | -0.01 | 3.22 | -6.43 | 0.80 | 0.12 | -0.57 | 0.33 | -32.27 | 2.57 | -160.30 | -0.01 | -157.41 | 96.76 | -4.90 | -4.44 | -25.89 | -0.31 |
| ***C.acnes* "high" (n = 6-8)** | Median | 0.00 | -0.23 | 0.49 | 0.00 | -0.03 | 8.11 | 0.06 | 0.05 | 0.00 | -0.05 | 117.03 | -0.55 | 42.26 | 0.46 | 32.98 | 8.29 | 4.10 | -0.60 | 17.66 | -0.02 |
|  | 1. Quartile | -0.01 | -1.21 | 0.41 | -0.03 | -1.12 | 1.85 | 0.04 | -0.04 | -1.19 | -0.29 | -2.01 | -1.73 | 26.78 | 0.00 | -12.00 | -12.14 | 2.71 | -1.73 | 1.34 | -0.10 |
|  | 3. Quartile | 0.03 | 0.26 | 0.89 | 0.01 | 0.48 | 26.06 | 0.29 | 0.16 | 0.28 | 0.17 | 463.19 | 0.98 | 232.90 | 1.20 | 175.08 | 41.86 | 8.53 | 2.96 | 32.26 | 0.31 |
| ***C. acnes*  "low" vs. "high"** | FDR  q-value | 0.99 | 0.34 | 0.13 | 0.21 | 0.41 | 0.01 | 0.99 | 0.84 | 0.57 | 0.68 | 0.07 | 0.07 | 0.01 | 0.13 | 0.01 | >0.99 | 0.01 | 0.01 | 0.01 | 0.13 |

**Supplementary Table 5:** Correlation of intradiscal *C.acnes* copies with Δ_MC1-control_ BM plasma cytokine levels.

|  | **GM-CSF** | **IFN-y** | **IL-1⁠β⁠** | **IL-4** | **IL-6** | **IL-8** | **IL-10** | **IL-12-p70** | **IL-13** | **IL-17A** | **ENA-78** | **G-CSF** | **IL-18** | **IL-7** | **IP-10** | **MCP-1** | **M-CSF** | **MIP-1a** | **MIP-1β⁠** | **TNF-α** |
| --- | --- | --- | --- | --- | --- | --- | --- | --- | --- | --- | --- | --- | --- | --- | --- | --- | --- | --- | --- | --- |
| Spearman r | 0.12 | -0.42 | 0.61 | 0.53 | -0.46 | 0.72 | -0.17 | -0.30 | 0.19 | -0.35 | 0.43 | -0.70 | 0.78 | 0.34 | 0.74 | -0.14 | 0.74 | 0.79 | 0.77 | 0.30 |
| Number of XY Pairs | 13 | 9 | 13 | 13 | 13 | 13 | 9 | 9 | 13 | 9 | 13 | 13 | 13 | 13 | 13 | 13 | 13 | 13 | 13 | 13 |
| P-value | 0.71 | 0.16 | 0.04 | 0.06 | 0.12 | 0.01 | 0.68 | 0.44 | 0.53 | 0.36 | 0.14 | 0.01 | 0.00 | 0.26 | 0.01 | 0.66 | 0.01 | 0.00 | 0.00 | 0.32 |

**Supplementary Table 6:** Blood plasma cytokine profiles of *C.acnes* “low” and “high” MC1 patients. MC1 patients of “low” group: n=10; MC1 patients of “high” group: n=9.

|  |  | **GM-CSF** | **IFN-y** | **IL-1⁠β⁠** | **IL-4** | **IL-6** | **IL-8** | **IL-10** | **IL-12-p70** | **IL-13** | **IL-17A** | **ENA-78** | **G-CSF** | **IL-18** | **IL-7** | **IP-10** | **MCP-1** | **M-CSF** | **MIP-1a** | **MIP-1β⁠** | **TNF-α** |
| --- | --- | --- | --- | --- | --- | --- | --- | --- | --- | --- | --- | --- | --- | --- | --- | --- | --- | --- | --- | --- | --- |
| **C. acnes "low" (n = 10)** | Median | 0.07 | 40.84 | 0.00 | 0.03 | 2.87 | 11.66 | 0.61 | 0.37 | 1.84 | 2.78 | 1162.71 | 10.85 | 396.46 | 7.77 | 473.33 | 220.03 | 12.18 | 31.93 | 101.90 | 2.70 |
|  | 1. Quartile | 0.00 | 30.61 | 0.00 | 0.01 | 1.72 | 7.33 | 0.22 | 0.24 | 1.25 | 1.08 | 559.69 | 9.04 | 364.90 | 6.48 | 406.41 | 196.85 | 10.37 | 29.85 | 79.70 | 2.48 |
|  | 3. Quartile | 0.09 | 57.93 | 0.41 | 0.07 | 5.62 | 18.03 | 0.90 | 0.48 | 3.15 | 4.16 | 2150.92 | 13.28 | 475.32 | 9.79 | 687.80 | 260.38 | 15.36 | 33.41 | 116.82 | 3.35 |
| **C. acnes "high" (n = 9)** | Median | 0.00 | 19.06 | 0.00 | 0.03 | 1.17 | 8.96 | 0.19 | 0.14 | 0.00 | 1.02 | 842.97 | 9.35 | 440.27 | 3.62 | 463.10 | 224.71 | 10.97 | 25.80 | 45.21 | 1.87 |
|  | 1. Quartile | 0.00 | 16.10 | 0.00 | 0.00 | 0.69 | 5.33 | 0.15 | 0.05 | 0.00 | 0.34 | 542.01 | 8.94 | 389.25 | 1.44 | 308.15 | 171.43 | 10.47 | 18.06 | 37.63 | 1.48 |
|  | 3. Quartile | 0.02 | 24.34 | 0.00 | 0.05 | 1.77 | 10.81 | 0.33 | 0.21 | 0.00 | 2.61 | 2019.57 | 10.69 | 486.55 | 7.08 | 545.37 | 270.85 | 13.62 | 28.79 | 106.28 | 2.56 |
| **C. acnes  "low" vs. "high"** | FDR q-value | 0.31 | 0.13 | 0.76 | 0.69 | 0.24 | 0.30 | 0.24 | 0.14 | 0.02 | 0.40 | 0.84 | 0.69 | 0.84 | 0.18 | 0.62 | 0.84 | 0.69 | 0.14 | 0.31 | 0.14 |

**Supplementary Table 7:** Correlation of intradiscal *C.acnes* copies with blood plasma cytokine levels.

|  | **GM-CSF** | **IFN-y** | **IL-1⁠β⁠** | **IL-4** | **IL-6** | **IL-8** | **IL-10** | **IL-12-p70** | **IL-13** | **IL-17A** | **ENA-78** | **G-CSF** | **IL-18** | **IL-7** | **IP-10** | **MCP-1** | **M-CSF** | **MIP-1a** | **MIP-1β⁠** | **TNF-α** |
| --- | --- | --- | --- | --- | --- | --- | --- | --- | --- | --- | --- | --- | --- | --- | --- | --- | --- | --- | --- | --- |
| Spearman r | -0.18 | -0.64 | -0.09 | -0.14 | -0.37 | -0.38 | -0.37 | -0.54 | -0.71 | -0.25 | -0.19 | -0.18 | 0.19 | -0.43 | -0.07 | -0.01 | -0.03 | -0.43 | -0.32 | -0.29 |
| Number of XY Pairs | 19 | 19 | 19 | 19 | 19 | 19 | 19 | 19 | 19 | 19 | 19 | 19 | 19 | 19 | 19 | 19 | 19 | 19 | 19 | 19 |
| P-value | 0.46 | 0.00 | 0.73 | 0.58 | 0.12 | 0.10 | 0.12 | 0.02 | 0.00 | 0.31 | 0.43 | 0.47 | 0.44 | 0.07 | 0.78 | 0.95 | 0.91 | 0.06 | 0.18 | 0.22 |

**Supplementary methods**

The study was conducted in accordance with the Declaration of Helsinki and approved by the local Ethics Commission (BASEC 2017-00761). Chemicals were purchased from Sigma-Aldrich, Buchs, Switzerland, if not stated otherwise.

Bulk RNA sequencing

The library of total cells was prepared with an input of 1 ug total RNA using the TruSeq®Stranded mRNA preparation kit (Illumina). Libraries were sequenced using the NovaSeq 6000 sequencer (Illumina) in single read mode 101 cycles and generating > 20 million reads per sample. The quality of data readings was assessed using FastQC. Adaptor sequences located at the 3’ ends were removed, and 4 bases were trimmed from each end using Trimmomatic (v0.36). Only readings with a length of > 30 nt were analyzed. These readings were aligned to the reference genome hg38 using STAR (v2.6.0c) and counted with FeatureCounts function in the Rsubreads package. Data is available at the European Nucleotide Archive at EMBL-EBI under accession number PRJEB61717. Differential expression analysis comparing MC1 to intra-patient controls was performed with DESeq2 (v3.16), using patient as a secondary factor. To identify transcriptomic changes that differed most between C.acnes “low” and “high” groups, gene expression of MC1 was first normalized to intra-patient controls and then analyzed with DEseq2. Genes were differentially expressed (DEGs) for p < 0.01. Bioinformatic overrepresentation analysis (ORA) was performed using the R package cluster profiler (v 4.6.2) with the identified DEGs and using the enrichment analysis tool Enrichr60. Terms were significantly overrepresented if false discovery rate (FDR) was < 0.05. Gene set enrichment analysis (GSEA) was performed using the R package cluster profiler (v 4.6.2) and with Web-based GSEA Toolkit (WebGestalt)61 with genes ranked by log2 fold change ratio. Gene sets were significantly overrepresented if FDR was < 0.05.

Cytokines measured in blood and BM plasma

Total protein concentration (pg/ml) of granulocyte colony stimulating factor (G-CSF), granulocyte-macrophage colony stimulating factor (GM-CSF), interferon gamma (IFN-y), interferon gamma-induced protein 10 (IP-10), interleukin 1β (IL-1β), IL-4, IL-6, IL-7, IL-8, IL-10, IL-12-p70, IL-13, IL-17A, IL-18, macrophage colony stimulating factor (M-CSF), macrophage inflammatory protein 1 (MIP-1α), MIP-1β, monocyte chemoattractant protein (MCP-1), neutrophil activating peptide (ENA-78), tumor necrosis factor alpha (TNF-α) of BM and blood plasma was measured in duplicates with MesoScale U-Plex (Meso Scale Diagnostics).
